# Molecular Image-Guided Theranostic and Personalized Medicine 2013

**DOI:** 10.1155/2014/682527

**Published:** 2014-05-29

**Authors:** Hong Zhang, Mei Tian, Ignasi Carrio, Ali Cahid Civelek, Yasuhisa Fujibayashi

**Affiliations:** ^1^The Second Affiliated Hospital of Zhejiang University, Hangzhou, Zhejiang 310009, China; ^2^Hangzhou Binjiang Hospital of Zhejiang University, Hangzhou, Zhejiang 310009, China; ^3^San Paul Hospital, Autonomous University of Barcelona, 08025 Barcelona, Spain; ^4^University of Louisville Medical Center, Louisville, KY 40202, USA; ^5^National Institute of Radiological Sciences, Chiba 263-8555, Japan

Molecular imaging differs from traditional imaging in that imaging agents or probes enable the visualization of the cellular or molecular function and followup of the molecular process in living subjects. It has been shown to be an effective noninvasive technology on improving diagnosis, prognosis, planning, and monitoring of personalized medication. Molecular imaging is a diverse technology which includes various modalities, for instance, positron emission tomography (PET), single photon emission computed tomography (SPECT), magnetic resonance imaging (MRI), computed tomography (CT), ultrasound (US), and optical imaging (Raman, quantum dots, bioluminescence, photoacoustic imaging, etc.). In the era of personalized theranostics (therapeutics and diagnostics), molecular imaging with the specific probe(s) would allow the clinical physicians to select the right patients for the appropriate treatment.

Topics covered in this special issue include advances in biomarkers in preclinical drug discovery, molecular imaging modalities in disease management, image-guided therapy approach of diseases, and imaging technology in drug development. For example, Dr. Z. Cheng's group at Stanford University reported an Affibody molecule, Ac-Cys-Z_EGFR:1907_, targeting the extracellular domain of epidermal growth factor receptor (EGFR), which was newly developed and used for detection of hepatocellular carcinoma (HCC). They observed that EGFR-expressing HCC lesions could be specifically detected by Ac-Cys-Z_EGFR:1907_. PET imaging might have better diagnostic value than optical imaging based on the same Affibody. The early and sensitive detection of HCC based on molecular cancer markers, such as EGFR, is a critical step in improving the currently dismal prognosis of HCC patients. Dr. H. Ma et al. presented a comparison study on ^99m^Tc-N-DBODC5 and ^99m^Tc-MIBI in patients with coronary artery disease. Their study results demonstrated that ^99m^Tc-N-DBODC5 and ^99m^Tc-MIBI MPI provide comparable diagnostic information for patients undergoing exercise rest for detection of CAD. In addition, ^99m^Tc-N-DBODC5 does not exhibit the disadvantages of ^99m^Tc-MIBI in their study.

Stem cell therapy is a promising therapeutic approach for some human major diseases. Molecular imaging plays an important role in terms of noninvasive, in vivo trafficking and evaluation of short-term or long-term therapeutic response. In this issue, molecular imaging in stem cell therapy for spinal cord injury and stroke were reviewed by F. Song et al. and F. Chao et al., who provided up-to-date information and future perspectives on these hot topics. Recently, molecular imaging, especially PET, has been introduced to evaluate the functional or metabolic changes in cerebral ischemia after administration of traditional Chinese medicine. Most of these works were done by the group from the Second Hospital of Zhejiang University School of Medicine. Z. Wang et al. from the same group reviewed molecular imaging in applications of traditional Chinese medicine in neurological disease.

New technologies, such as microfluidics for synthesis of PET tracers and acoustic droplet vaporization, were included in this special issue. Over the last decade, microfluidics and lab-on-a-chip (LOC) technology have boomed as powerful tools in the field of organic chemistry, which potentially provides significant help to the PET chemistry. Microfluidic radiolabeling technology and its application for synthesis of peptide-based PET tracers are summarized and discussed in the review paper by Dr. Y. Liu et al. Acoustic droplet vaporization (ADV) is a physical process in which the pressure waves of ultrasound induce a phase transition that causes superheated liquid nanodroplets to form gas bubbles. The bubbles provide ultrasonic imaging contrast and other functions. C.-Y. Lin and W. G. Pitt reviewed the literature regarding the use of ADV in clinical applications of imaging, embolic therapy, and therapeutic delivery.

An interesting study on abacus training in Chinese kids using functional magnetic resonance imaging (fMRI) found that abacus training modulates the neural correlations of exact and approximate calculations. Additionally, findings on the influence of ^131^I radioablation on the important lymphocytes subtypes of regulatory T and B cells in patients with papillary thyroid carcinoma and the prognostic value of interim and final FDG-PET in major histotypes of B cell NHL patients treated with rituximab-containing chemotherapy have been discussed in this special issue.

In summary, molecular imaging could be applied to target characterization, underlying disease progression, and evaluation of therapeutic response after administration of western medication, stem cell, and traditional medicine. This special issue provides a platform of efficacy of personalized medication from molecular imaging technology which may have high impact on patient care and drug development.

*Hong Zhang* *Mei Tian* *Ignasi Carrio* *Ali Cahid Civelek* *Yasuhisa Fujibayashi*

